# Capturing protein-protein interactions in plants: recent advances, challenges, and opportunities

**DOI:** 10.3389/fmolb.2026.1777595

**Published:** 2026-03-06

**Authors:** Berry Dickey, Yatendra Singh, Sibesh Maharjan, Yongjian Qiu, Sixue Chen

**Affiliations:** Department of Biology, University of Mississippi, Oxford, MS, United States

**Keywords:** AlphaFold, mass spectrometry, plants, protein-protein interactions, proximity labeling

## Abstract

Plants rely on dynamic protein-protein interaction (PPI) networks to carry out routine functions (such as photosynthesis and respiration) and responses to environmental cues. Therefore, capturing dynamic PPIs is critical for understanding molecular processes underlying the plant life cycle. Recent technological advances have significantly expanded the experimental and computational toolkit available for studying PPIs in plants. In this review, emerging and advanced technologies are presented, including proximity labeling, yeast-3-hybrid, AlphaFold 3, and data-independent acquisition mass spectrometry (MS). How these technologies address critical limitations posed by classical techniques, and their strengths, challenges, and opportunities, are discussed. The goal is to provide an updated practical guide that informs researchers on selecting, optimizing, and combining these tools to maximize protein interactome coverage. Together, these complementary tools and approaches promise to advance mechanistic understanding of plant biological processes and enable more informed manipulation of complex biological networks toward improving crop quality and yield.

## 1 Introduction

Plants are sessile organisms that must sense and respond to biotic and abiotic factors to survive, adapt, and reproduce. Proteins orchestrate diverse pathways within or between individual cells to coordinate how tissues and the whole plant function. Compared with other kingdoms, plants as a whole have the largest range of genome sizes and, consequently, the most diverse proteome. The reference plant *Arabidopsis thaliana* contains approximately 27,500 protein-coding genes, whereas a plant with a larger genome, like *Oryza sativa* (rice), contains approximately 35,500 protein-coding genes (Cheng et al., 2017; Jain et al., 2019). A large-scale proteomic survey covering 13 plant species, including model plants and crops, identified over 141,000 unique proteins utilizing mass spectrometry (MS)-based methodologies (McWhite et al., 2020). Another study reported that an *Arabidopsis* root cell contains, on average, 170 pg of protein (Clark et al., 2022; Montes et al., 2024), which corresponds to approximately 2.2 billion protein molecules. An *Arabidopsis* mesophyll cell was estimated to contain about 25 billion protein molecules (Heinemann et al., 2021). These protein molecules do not act alone; they often interact with each other and with other types of molecules.

Plants rely on dynamic protein-protein interactions (PPIs) to translate environmental cues into physiological and developmental responses. For instance, proteins can assemble into transcriptional regulatory complexes that activate genes involved in a specific response. When these target genes encode enzymes for phytohormone biosynthesis (e.g., auxin), the resulting PPI-driven response, can promote adaptive growth, such as hypocotyl elongation in seedlings at moderately elevated temperatures (Gray et al., 1998; Qiu et al., 2020). Such cause-and-effect events occur rapidly across different cell types, collectively forming a highly coordinated system that enables plants to sense and respond to changing conditions. Drawing PPI models and connecting PPI networks on a large scale, especially in the context of stress responses, is vital to this age of plant biology research and agricultural applications.

**FIGURE 1:**
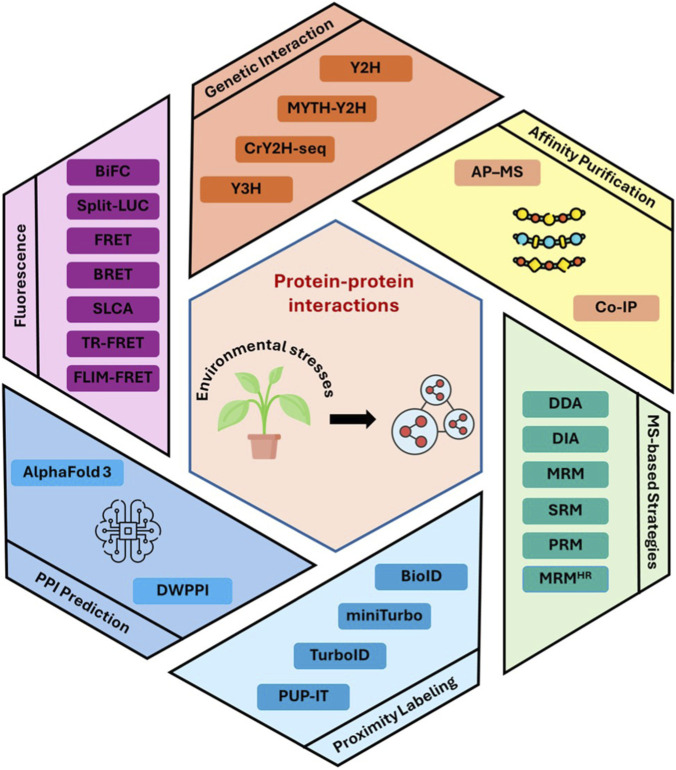
Schematic overview of experimental and computational approaches used to study protein-protein interactions (PPIs) in plants. Abbreviations: Y2H, yeast two-hybrid; Y3H, yeast three-hybrid; MYTH, membrane yeast two-hybrid; CrY2H-seq, Cre reporter-mediated yeast two-hybrid with sequencing; Co-IP, co-immunoprecipitation; AP-MS, affinity purification-mass spectrometry; SLCA, split-luciferase complementation assay; BiFC, bimolecular fluorescence complementation; FRET, Förster resonance energy transfer; BRET, bioluminescence resonance energy transfer; TR-FRET, time-resolved FRET; FLIM-FRET, fluorescence lifetime imaging microscopy-FRET; PUP-IT, pupylation-based interaction tagging; DDA, data-dependent acquisition; DIA, data-independent acquisition; MRM, multiple reaction monitoring; MRM^HR^, multiple reaction monitoring at high resolution; SRM, selected reaction monitoring; PRM, parallel reaction monitoring; DWPPI, deep learning approach for predicting PPIs in plants based on multi-source information.

In this review, we summarize widely used and newly emerging methods for the prediction, identification, and validation of PPIs. Traditional experimental approaches that have been heavily utilized within previous decades are briefly described, followed by discussions about advancements of new technologies in mapping plant PPIs (Figure 1; Table 1). The main objective is not to review the field in its entirety, but rather to focus on technologies and their improvements, limitations, and future opportunities.

**TABLE 1:** Comparative overview of classical and emerging PPI technologies in plants.

Methodology	Method category	Interaction context	Key strengths	Major limitations	Applications in plants
Y2H	Genetic interaction system	Interaction in the yeast nucleus	Simple, fast, low cost	High false positives; interaction restricted to the nucleus	Binary interaction screening
MYTH		Interaction on the membrane	Enables membrane PPI detection	Limited protein classes	Membrane interactomics
Y3H		Interaction in the yeast nucleus	Ternary or ligand-mediated PPIs	Complex setup; indirect interactions	PTM- or metabolite-dependent PPIs
CrY2H-seq			Multiplexed and high throughput interactome mapping	Still binary, yeast-specific context	Large-scale transcription factor and regulator interactomes
Co-IP	Affinity-based	*ex vivo*	More reliable than Y2H at detecting native PPIs	Loses transient interactions Lysis-derived false positives	Validation of known PPIs
AP-MS	Affinity-based + MS		Detect novel protein interactors through LC-MS/MS		Large-scale interactome discovery
BiFC	Fluorescence in living cells	*In vivo*	Visual localization in real time	Inducing irreversible complex formation	Subcellular PPI validation
SLCA	Luminescence signal intensity		Quantitative, dynamic	Requires an endogenous supply of luciferin	Real-time PPI visualization
FRET BRET	Energy-transfer		Kinetic and stoichiometric data	Spectral crosstalk and excitation can introduce false positives	
BioID	PL + MS	*In vivo →* *ex vivo*	Captures proximal and transient PPIs	Slow labeling; background biotinylation	Stable interactome profiling
TurboID			Rapid labeling (∼10 min)	High background	Stress- and signal-induced PPIs
miniTurbo			Lower background than TurboID	Reduced sensitivity	Cell-type-specific PPIs
PUP-IT			High specificity, little background, no exogenous substrate addition	Large size may restrict some interaction sites	High confidence PPIs Captures dynamic interactions
AlphaFold3	Computational	*in silico* (multi-molecular complexes)	Integrated prediction of PPIs, protein-DNA/RNA, and protein-ligand interactions	Limited accuracy for highly dynamic systems	Modeling regulatory and multi-component complexes

Abbreviation: Y2H, yeast two-hybrid; MYTH, membrane Y2H; Y3H, yeast three-hybrid; CrY2H-seq, cre reporter-mediated Y2H system combined with next-generation sequencing; Co-IP, co-immunoprecipitation; AP-MS, affinity purification-mass spectrometry; SLCA, split-luciferase complementation assay; BiFC, bimolecular fluorescence complementation; FRET, forster resonance energy transfer; BRET, bioluminescence resonance energy transfer; PUP-IT, pupylation-based interaction tagging; PL, proximity labeling.

## 2 Classic technologies

### 2.1 Yeast two-hybrid and three-hybrid

Yeast two-hybrid (Y2H) system was established in 1989 (Fields and Song, 1989) as a pioneering method for studying PPIs in the yeast nucleus. Y2H relies on fusing two proteins of interest (bait and prey) to the DNA-binding domain (BD) and the activation domain (AD) of a yeast transcriptional activator, respectively. Upon interaction of the proteins, the transcription factor is reconstituted, driving expression of a reporter gene that enables detection of the interaction (Zhang et al., 2010). The assay is simple, fast, and low-cost, contributing to its long-standing utility. However, it exhibits several limitations, including a relatively high false-positive rate, restriction of detectable interactions to the nucleus, potential toxicity of certain proteins in yeast (resulting in false negatives), and its inherently binary nature. Thus, interactions detected in yeast typically require independent validation *in planta* (Struk et al., 2019). Several improved Y2H derivatives have been developed. For example, a split-ubiquitin-based membrane Y2H (MYTH) system adapts the classical concept to the membrane environment by splitting ubiquitin into two fragments fused to separate proteins (Miller et al., 2005; Struk et al., 2019). The MYTH system is particularly advantageous for studying interactions of hydrophobic or membrane-associated proteins. To substantially increase screening capacity, a multiplexed Cre reporter-mediated Y2H system combined with next-generation sequencing (CrY2H-seq) was developed to enable deep coverage and high-throughput mapping of large interactomes (Wanamaker et al., 2017). Application of CrY2H-seq screening to transcription factors and regulators in *Arabidopsis* revealed approximately 8,500 binary interactions. It has become a powerful tool for large-scale interactome discovery, and it remains cost-effective and time-efficient (Table 1).

Because of the binary nature of the classical Y2H, a yeast three-hybrid (Y3H) system was developed. In Y3H, a third molecule, such as a protein, RNA, or metabolite, acts as a bridge to stabilize or mediate interactions between the bait and prey (Maruta et al., 2016). This system enables the analysis of protein-RNA and protein-small molecule interactions, identification of post-translational modification (PTM)-dependent interactions, and screening for inhibitors that disrupt PPIs (Table 1).

### 2.2 Co-immunoprecipitation and affinity purification-mass spectrometry

Co-immunoprecipitation (Co-IP) and affinity purification-mass spectrometry (AP-MS) are two traditional approaches used to capture *in vivo* PPIs (Zhang et al., 2010; Zhang et al., 2022). Both methods are considered more reliable than Y2H for detecting native PPIs because they can be directly implemented in plant systems, such as through leaf infiltration with *Nicotiana benthamiana* or stable transformation in Arabidopsis (Zhang et al., 2019; Gnanasekaran and Pappu, 2023). Co-IP and AP-MS generally require a detectable bait protein (with or without an affinity tag) in plant tissues or protoplasts, through which endogenous protein interactors (prey) can be captured and analyzed. While AP-MS is a discovery-based system that can detect novel protein interactors through liquid chromatography-tandem MS (LC-MS/MS) of separated protein complexes, Co-IP is a targeted method that detects the interaction between known protein pairs through the use of specific antibodies (Zhang et al., 2022). This means that Co-IP results are traditionally detected via Western blot analysis. Major drawbacks for both methods include that cell lysis can generate false positives due to disrupting the native cellular compartmentalization and nonspecific interactions. False negatives can result from its inability to efficiently capture weak or transient interactions or from the disruption of these interactions during sample preparation and purification (Zhang et al., 2010). *In vivo* cross-linking is a way to stabilize PPIs and capture transient and unstable interactions (Chen et al., 2025; Trinh et al., 2025). Even though Co-IP and AP-MS capture PPIs *in vivo*, the actual detection of interactions is conducted through *in vitro* experiments, so both are considered *ex vivo* methods (Xing et al., 2016).

### 2.3 Advanced fluorescence techniques

While Co-IP and fixed-sample imaging, such as immunofluorescence (IF), are limited in capturing dynamic interactions, live-cell fluorescence methods enable real-time and high-resolution visualization of PPIs (Ren et al., 2024). Bimolecular fluorescence complementation (BiFC) and split-luciferase complementation assay (SLCA) are popular *in vivo* assays used to determine PPIs by combining two halves of a reporter (e.g., yellow fluorescent protein (YFP) or luciferase (Luc)) upon their interaction (Xing et al., 2016). BiFC detects PPIs through fluorescence imaging in living cells, whereas SLCA can provide quantitative measurements of PPI strength based on luminescent signal intensity. The major advantage of both methods is that the PPIs can be monitored visually in real-time in near-native cellular contexts. However, both approaches face limitations, including that BiFC induces irreversible complex formation and that SLCA requires exogenous supplementation of luciferin. Additionally, fusions of large proteins or reporter fragments may alter protein folding or hinder interactions, which could potentially lead to both false positives and negatives in these experiments.

For truly quantitative and kinetic analysis, Fluorescence Resonance Energy Transfer (FRET) has become the preferred technique (Zhang et al., 2025). It is a distance-dependent energy transfer process (nonradiatively) from an excited molecular fluorophore (the donor) to another fluorophore (the acceptor) through intermolecular dipole–dipole coupling. The FRET assay can reliably detect protein proximity when proteins are at distances of 10–100 Å and is highly efficient if they are within the Förster radius for the specific fluorophore pair (Sekar and Periasamy, 2003). FRET and its modern variants offer distinct advantages in quantifying interaction stoichiometry and kinetics. Further advancements, e.g., Time-Resolved FRET (TR-FRET) and Fluorescence Lifetime Imaging Microscopy-FRET (FLIM-FRET), provide superior quantitative data essential for detailed mechanistic studies of dynamic PPIs (Matthes et al., 2025; Zhang et al., 2025).

Although FLIM-FRET has been used to study plant PPIs, its application under native conditions is limited by strong autofluorescence and insufficient donor brightness, particularly for low-abundant proteins expressed from endogenous promoters and localized to challenging subcellular compartments such as the plasma membrane (Donaldson, 2020). Plant autofluorescence can overlap with the emission spectra of commonly used fluorophores, thereby reducing signal-to-noise ratios and complicating the extraction of accurate fluorescence lifetime information. In addition, fluorescence lifetimes can be affected by local environmental factors (e.g., pH, temperature, and refractive index variations within the vacuole or apoplast), which may result in false-positives or false-negatives (Eljebbawi et al., 2025). A recent study overcame some of these constraints by introducing an optimized fluorescent protein pair, mCitrine/mScarlet-I, which provides superior brightness and fluorescence lifetimes compatible with FLIM-FRET in stably transformed *Arabidopsis* (Petutschnig et al., 2024). This improved FLIM-FRET system enabled direct detection of constitutive and ligand-induced interactions between the immune receptors CERK1 and LYK5 in *Arabidopsis* at endogenous expression levels.

Bioluminescence Resonance Energy Transfer (BRET) is an alternative proximity-based assay where the donor fluorophore is replaced by a bioluminescent protein, typically a luciferase. Upon the addition of a substrate (e.g., coelenterazine), the luciferase-produced light excites a nearby acceptor fluorophore. A key limitation of BRET is the requirement for continuous or repeated substrate addition, which may restrict long-term imaging and cause toxicity in sensitive plant cells (El Khamlichi et al., 2019). Additionally, uneven substrate penetration and distribution across the cell wall and plasma membrane can introduce variability. BRET is generally less suited for capturing rapid dynamic interactions. Moreover, the signal intensity is often lower than that achieved with fluorescence-based methods due to the kinetics of bioluminescent reactions, reducing sensitivity for detecting low abundant or transient PPIs. Despite these limitations, BRET offers distinct advantages over FLIM-FRET for plant PPI studies. Because it does not rely on external excitation, BRET is unaffected by chlorophyll autofluorescence, resulting in a substantially higher signal-to-noise ratio in green tissues (Sun et al., 2016). Also, the absence of high-intensity laser illumination makes BRET less phototoxic, enabling gentle and close-to-native monitoring of PPIs during plant development or stress responses.

## 3 Emerging and improving technologies

### 3.1 Proximity labeling

Proximity labeling (PL) has emerged as a transformative research approach for capturing PPIs in plants, addressing many of the inherent limitations offered by classical methods (Struk et al., 2019; Özmen et al., 2025). Unlike traditional affinity purification techniques, where PPIs are captured for analysis after cell lysis, PL utilizes engineered enzymes (e.g., biotin ligase) to covalently tag neighboring proteins (e.g., biotin) in living cells, enabling subsequent analysis using harsh methods without disrupting the tag and minimizing false positive results. For complex, dynamic, and/or temporal protein networks, such as those involved in regulatory mechanisms or high-flux pathways in plant stress responses, PL methods enable the unprecedented large-scale discovery of novel stable and transient protein interactions *in vivo* when followed by tandem MS analysis (Table 1).

#### 3.1.1 Biotin ligase systems

Biotin ligase-based PL was first introduced in mammalian cells in 2012 with the development of BioID, a promiscuous mutant (R118G) of the *Escherichia coli* biotin ligase BirA (35 kDa) fused to a bait protein (Choi-Rhee et al., 2004; Roux et al., 2012). BioID2, a smaller biotin ligase (27 kDa) derived from *Aquifex aeolicus*, was later developed to reduce the steric burden associated with the larger BirA (Kim et al., 2016). Following exogenous biotin incubation, protein biotinylated within proximity of the bait can be enriched with streptavidin beads and analyzed by LC-MS/MS (Mair et al., 2019; Özmen et al., 2025). BioID has been applied to different organisms (e.g., yeast, *Drosophila*, zebrafish, mice, and plants), but the biotinylation activity is low and can take up to 24 h for labeling (Larochelle et al., 2019; Mair et al., 2019; Takano et al., 2020; Rosenthal et al., 2021; Zhang et al., 2021; Özmen et al., 2025).

To overcome this limitation, TurboID (TbID) and miniTurbo (mTb) were developed to provide dramatically faster labeling kinetics, achieving robust biotinylation within minutes rather than hours (Branon et al., 2018). They were rapidly adopted in *Arabidopsis* and *N. benthamiana* (Branon et al., 2018; Khan et al., 2018; Mair et al., 2019). TbID exhibits higher activity than mTb in stably transformed plant tissues. The applications of TbID and mTb are widespread for PL of interactomes in plants (Zhang et al., 2022; Özmen et al., 2025), and they outperform the original BioID (Feng et al., 2024; Park and Kim, 2025). However, a major limitation of the TurboID-based systems is elevated background biotinylation, which reduces specificity and complicates data interpretation. This background arises from TurboID’s high catalytic activity, leading to non-specific labeling, as well as diffusion of the reactive biotinyl-AMP intermediate that creates a local “cloud” of off-target biotinylation. In addition, endogenously biotinylated proteins and high levels of endogenous biotin contribute to the background signal. Compared with BioID, TbID provides much faster labeling but at the cost of increased background, whereas mTb partially alleviates this issue by lowering the catalytic activity while retaining acceptable labeling efficiency. To mitigate background biotinylation, several strategies are employed, e.g., shortening labeling times, optimizing biotin concentrations to the lowest effective levels, and using genetic approaches to decrease endogenous biotin synthesis. Split TurboID systems and optogenetically controlled variants, such as OptoID, allow for the conditional activation of biotinylation, thereby improving spatial and temporal precision (Chen et al., 2022). Despite these limitations, optimized TurboID systems remain powerful and versatile tools for PL proteomics across various biological systems.

#### 3.1.2 Pupylation-based interaction tagging

A different method of PL utilizing pupylation-based interaction tagging (PUP-IT) was first demonstrated in mammalian T cells (Liu et al., 2018). Pupylation is facilitated by the bacterial PafA enzyme, which tags lysine residues on target proteins with a small protein called Pup(E), marking those proteins for degradation, similar to ubiquitination. Pup(E) is tightly bound with PafA, and its release for tagging requires proteins to interact within close proximity (Liu et al., 2018). This lends PUP-IT systems inherent specificity in labeling and decreased sensitivity to distal protein interactions. In plants, PL with PUP-IT offers further advantages over biotin ligase-based systems, as pupylation is not endogenous to eukaryotes and does not require exogenous substrate addition, resulting in low background and simplified workflows (Ye et al., 2023; Özmen et al., 2025).

Recent applications have demonstrated the utility of the PUP-IT system. For example, it was used with tandem MS to identify kinase substrates in *Arabidopsis* protoplasts, demonstrating its versatility in cell suspensions and in capturing dynamic interactions in signaling pathways, like phosphorylation of target substrates (Ye et al., 2023). Recently, PUP-IT was implemented both transiently in *N. benthamiana* and in *Arabidopsis* stable transgenic lines to identify interactors of the cellulose synthase complex (CSC), showing compatibility with diverse bait proteins, single-transformation applications, and customizable affinity purification strategies (e.g., Flag-tagged PupE) (Zheng et al., 2025). Using COMPANION OF CESA 1 (CC1), one of the central CSC components, as bait, Persson’s group successfully enriched other core CSC components and trafficking regulators. Beyond validating known interactions, PUP-IT enabled the identification of BFA-VISUALIZED ENDOCYTIC TRAFFICKING DEFECTIVE1 (BEN1), an ADP-ribosylation factor guanine nucleotide exchange factor (ARF-GEF) involved in trans-Golgi network trafficking, as a previously unrecognized CC1-associated protein. Subsequent biochemical and cell biological analyses confirmed CC1-BEN1 interaction and revealed BEN1 poly-ubiquitination, illustrating how PUP-IT can uncover novel, functionally relevant interactors of membrane-embedded protein complexes in plants. Moreover, bait-interactor substrates were more enriched with pupylation through PUP-IT than with biotinylation through TbID, suggesting PUP-IT may have higher specificity in its labeling (Zheng et al., 2025).

Despite its strengths, PUP-IT has several limitations. First, the relatively large size of the pupylation components (∼50 kDa) may restrict access to certain interaction sites (Yue et al., 2022). Second, Pup(E) has limited diffusion across membranes, reducing its suitability for certain organelles. Third, overexpression-based assays may increase non-specific labeling. Fourth, Pup(E) expression or accessibility may vary across tissues. Finally, when inducible promoters are used, leaky background expression or delayed activation may occur. These considerations highlight the need for careful optimization and appropriate controls when applying PUP-IT in plant systems.

### 3.2 Structural prediction and network modeling

The vast scale of the proteome and the challenges inherent in experimental validation have driven computational methods, artificial intelligence (AI), and its subset, deep learning (DL), to predict and map PPIs (Ding and Kihara, 2018; Lee, 2023). In 2020, AlphaFold was released for protein structure prediction (Senior et al., 2020) and subsequently, AlphaFold2 further advanced its ability to address the long-standing protein-folding problem (Jumper et al., 2021). AlphaFold-Multimer was later introduced, specifically trained to predict protein complexes (Evans et al., 2022). It enables computational exploration of PPIs, oligomeric states, and assembly structures that are critical for understanding signaling pathways, molecular machines, and multi-protein complexes in cells. AlphaFold3 (AF3) further expanded the modeling framework beyond proteins to include DNA, RNA, PPIs, and a wide range of molecular interactions. However, it is noteworthy that AF3 is still facing challenges in predicting interactions involving intrinsically disordered regions (IDRs) in proteins and proteins remain folded within a large, multi-subunit complex (Wee and Wei, 2024). AF3 also struggles with large or plant-specific multimeric complexes (e.g., immune signaling or photosynthetic assemblies), environmental dependencies, limited evolutionary data for unknown proteins, often leading to false positives that require experimental validation (e.g., via biophysical interaction assays) to confirm relevance for plant PPIs. Additionally, specific PTMs (e.g., unique glycosylation patterns) are yet to be integrated into AF3’s interaction modeling.

Recent frameworks (e.g., ABCFold) combine AF3 prediction with comparable structure-oriented models (e.g., Boltz-1 and Chai-1) to enhance robustness and confidence in predicted protein complexes (Elliott et al., 2025). In addition to structure-based approaches, sequence-based predictors have also been widely developed to infer PPIs at scale. Representative DL models, including D-SCRIPT and PIPR, predict interaction likelihood directly from amino acid sequences without requiring explicit structural information (Sledzieski et al., 2021; Chen et al., 2019). Notably, DWPPI was specifically designed for plant PPI prediction and integrates sequence-derived features with network-derived embeddings obtained from plant PPI graphs, thereby incorporating both protein attributes and topological context (Pan et al., 2022). This hybrid strategy improves predictive performance in plant systems, where experimentally validated PPI data are relatively sparse. The effectiveness of DWPPI has been demonstrated using multiple plant datasets, including *A. thaliana*, maize (*Zea mays*), and rice (*Oryza sativa*), achieving high prediction performance of PPIs (Pan et al., 2022).

In further development, protein language model-based approaches have advanced sequence-based prediction by leveraging embeddings pre-trained on massive protein sequence corpora, enabling improved generalization across species. Recent studies in plant systems have applied such pre-trained embeddings to predict *Arabidopsis* PPIs and plant-pathogen interaction networks, demonstrating improved performance and robustness despite the limited availability of experimentally validated plant PPI data (Lei et al., 2023; Zhou et al., 2023). In parallel, graph neural network (GNN) frameworks provide a complementary layer of network modeling by explicitly learning from PPI graph topology and integrating multi-source evidence, offering a systems-level approach to refine and contextualize predicted interaction networks (Yuan et al., 2022). We anticipate that AF3 and other DL models will continue to evolve at a fast pace and revolutionize different fields of research and application.

## 4 Mass spectrometry-based strategies

Data-dependent acquisition (DDA) and data-independent acquisition (DIA) are widely used MS approaches for studying PPIs. DDA selects and fragments the top 10–30 most intense precursor ions per cycle, providing confident identifications of interacting proteins. In contrast, DIA fragments all precursor ions within predefined m/z windows, offering comprehensive coverage and improved reproducibility (Fröhlich et al., 2024). In recent years, DIA proteomics has gained immense popularity owing to advances in fast MS duty cycles and AI-based prediction of MS^2^ spectra. In PPI analysis, size exclusion chromatography (SEC) combined with DDA-based quantitative proteomics has been used to characterize protein complexes in *Arabidopsis*, enabling the profiling of approximately 615 proteins as likely subunits of stable complexes including 338 cytosolic proteins (Aryal et al., 2017). While this study demonstrated the feasibility of SEC-based interactome profiling in plants, the proteome depth achievable with DDA remains limited compared with more recent SEC-DIA approaches. Indeed, SEC-DIA MS has shown power in resolving native protein complexes of 2,127 proteins in human cells (Heusel et al., 2019). To date, SEC-DIA MS for plant interactome studies has not been reported. Nevertheless, DIA-MS has been successfully applied in comprehensive plant protein identification and quantification (Sang et al., 2024; Rajczewski et al., 2025).

Although SEC-DIA MS is promising, its application to investigating large-scale plant PPIs involves trade-offs. SEC provides relatively low resolution; different complexes of similar hydrodynamic radii may co-elute, making it difficult to distinguish between distinct functional assemblies. Extracting intact, “native” protein complexes from plant cells often requires harsh physical disruption of cells (e.g., grinding in liquid nitrogen). This process may release phenolic compounds, redox molecules, and proteases that can alter complexes or interfere with SEC reproducibility and validity. The high abundance of RuBisCO in photosynthetic tissues can mask the signal of low-abundant signaling proteins, requiring depletion strategies that may inadvertently disrupt PPIs being studied. Additionally, DIA data analysis still faces challenges with multiplexed fragmentation data, quality of spectral libraries, and vast proteoforms with PTMs (Wen et al., 2025).

MS-based targeted quantification of peptides offers another approach for studying PPIs. Selected Reaction Monitoring (SRM), or Multiple Reaction Monitoring (MRM), utilizes a triple quadrupole (Q) mass spectrometer to monitor and isolate preselected precursor ions in Q1. The precursors are then fragmented in the Q2 collision cell, and specific product ions are measured in Q3. This dual-stage filtering provides high sensitivity quantification of low-abundant peptides (MacLean et al., 2010). Unlike SRM/MRM, parallel reaction monitoring (PRM) includes all fragment ions generated from the targeted precursor, providing greater specificity and quantitative accuracy. Recently, MRM^HR^, a high-resolution variant of MRM (similar to PRM), has also been very useful for protein quantification and validation in plants (Elmore et al., 2021; Tan et al., 2025). Together with DIA-MS, these targeted approaches allow highly selective, sensitive, and accurate quantification of specific protein partners, including low-abundance proteins, in complex samples.

## 5 Discussion

The landscape of PPI studies has undergone profound transformations driven by technological advances that have addressed the fundamental limitations of classical approaches (Figure 1; Table 1). The intrinsic limitations of early methods, such as the Y2H system, have necessitated methodological refinements and the development of new technologies. For example, extensive optimization and screening strategies have substantially improved Y2H reliability. False positives can be minimized by using multiple reporter genes driven by distinct promoters, which increases selection stringency and interaction specificity. Screening with diverse bait and prey vector configurations, including both N- and C-terminal fusions, further enhances robustness.

The progression from AP-MS to PL does not necessarily replace the older techniques. Rather, the toolkit for studying PPIs is ever-expanding, especially with improved deep-learning AI models like AF3 for protein complex predictions. While AF3 cannot replace experimental validation, it is exciting to see how it can be incorporated into PPI studies to enable more computationally informed preliminary experimental choices and downstream interaction validation.

Implementing AF3 will certainly influence the upstream decisions to prioritize targets in studying novel PPIs. On the other hand, PL techniques such as TurboID can first identify interactors, enabling AF3 to predict their interaction mechanisms. Moreover, SEC-DIA MS is expected to be expanded for use in plants to capture and quantify protein complexes. Collectively, the synergistic integration of optimized traditional assays, next-generation platforms, and AI-driven structural predictions represents a powerful framework for advancing PPI research in plants.

## 6 Concluding remark

Recent technological advances have broadened the PPI toolkit and enhanced knowledge acquisition. The future of PPI research lies in the thoughtful combination of optimized classical assays, next-generation PL technologies, and DL. Such integrated frameworks will not only enhance confidence in PPI discovery but also provide mechanistic insight into how protein networks coordinate plant growth, development, and response to stresses.

## References

[B1] Aryal U. K. Mcbride Z. Chen D. Xie J. Szymanski D. B. (2017). Analysis of protein complexes in Arabidopsis leaves using size exclusion chromatography and label-free protein correlation profiling. J. Proteomics 166, 8–18. 10.1016/j.jprot.2017.06.004 28627464

[B2] Branon T. C. Bosch J. A. Sanchez A. D. Udeshi N. D. Svinkina T. Carr S. A. (2018). Efficient proximity labeling in living cells and organisms with TurboID. Nat. Biotechnol. 36, 880–887. 10.1038/nbt.4201 30125270 PMC6126969

[B62] Chen M. Ju C. J.-T. Zhou G. Chen X. Zhang T. Chang K.-W. (2019). Multifaceted protein-protein interaction prediction based on Siamese residual RCNN. Bioinformatics 35, i305–i314. 10.1093/bioinformatics/btz328 31510705 PMC6681469

[B3] Chen R. Zhang N. Zhou Y. Jing J. (2022). Optical sensors and actuators for probing proximity-dependent biotinylation in living cells. Front. Cell. Neurosci. 16, 801644. 10.3389/fncel.2022.801644 35250484 PMC8890125

[B4] Chen J. Zhao Q. Zhang Y. Zhang L. (2025). *In vivo* cross-linking mass spectrometry: advances and challenges in decoding protein conformational dynamics and complex regulatory networks in living cells. Curr. Opin. Chem. Biol. 88, 102630. 10.1016/j.cbpa.2025.102630 40945469

[B5] Cheng C. Y. Krishnakumar V. Chan A. P. Thibaud‐Nissen F. Schobel S. Town C. D. (2017). Araport11: a complete reannotation of the *Arabidopsis thaliana* reference genome. Plant J. 89, 789–804. 10.1111/tpj.13415 27862469

[B6] Choi‐Rhee E. Schulman H. Cronan J. E. (2004). Promiscuous protein biotinylation by *Escherichia coli* biotin protein ligase. Protein Science 13, 3043–3050. 10.1110/ps.04911804 15459338 PMC2286582

[B7] Clark N. M. Elmore J. M. Walley J. W. (2022). To the proteome and beyond: advances in single-cell omics profiling for plant systems. Plant Physiol. 188, 726–737. 10.1093/plphys/kiab429 35235661 PMC8825333

[B8] Ding Z. Kihara D. (2018). Computational methods for predicting protein‐protein interactions using various protein features. Curr. Protoc. Protein Sci. 93, e62. 10.1002/cpps.62 29927082 PMC6097941

[B9] Donaldson L. (2020). Autofluorescence in plants. Molecules 25, 2393. 10.3390/molecules25102393 32455605 PMC7288016

[B10] El Khamlichi C. Reverchon-Assadi F. Hervouet-Coste N. Blot L. Reiter E. Morisset-Lopez S. (2019). Bioluminescence resonance energy transfer as a method to study protein-protein interactions: application to G protein coupled receptor biology. Molecules 24, 537. 10.3390/molecules24030537 30717191 PMC6384791

[B11] Eljebbawi A. Dolata A. Strotmann V. I. Weidtkamp-Peters S. Stahl Y. (2025). From principles to practice: a comprehensive guide to FRET-FLIM in plants. Methods Microsc. 2, 33–43. 10.1515/mim-2024-0019

[B12] Elliott L. G. Simpkin A. J. Rigden D. J. (2025). ABCFold: easier running and comparison of AlphaFold 3, Boltz-1 and Chai-1. Bioinforma. Adv. 5, vbaf153. 10.1093/bioadv/vbaf153 40708869 PMC12287924

[B13] Elmore J. M. Griffin B. D. Walley J. W. (2021). Advances in functional proteomics to study plant-pathogen interactions. Curr. Opin. Plant Biol. 63, 102061. 10.1016/j.pbi.2021.102061 34102449

[B14] Evans R. O’Neill M. Pritzel A. Antropova N. Senior A. Green T. (2022). Protein complex prediction with AlphaFold-multimer. BioRxiv. 10.1101/2021.10.04.463034

[B15] Feng L. Zhou J. Zhu D. Gao C. (2024). TurboID-based proximity labeling accelerates discovery of neighboring proteins in plants. Trends Plant Sci. 29, 383–384. 10.1016/j.tplants.2023.10.011 37949706

[B16] Fields S. Song O.-K. (1989). A novel genetic system to detect protein–protein interactions. Nature 340, 245–246. 10.1038/340245a0 2547163

[B17] Fröhlich K. Fahrner M. Brombacher E. Seredynska A. Maldacker M. Kreutz C. (2024). Data-independent acquisition: a milestone and prospect in clinical mass spectrometry–based proteomics. Mol. Cell. Proteomics 23, 100800. 10.1016/j.mcpro.2024.100800 38880244 PMC11380018

[B18] Gnanasekaran P. Pappu H. R. (2023). “Affinity purification-mass spectroscopy (AP-MS) and co-immunoprecipitation (Co-IP) technique to study protein–protein interactions,” in Protein-protein interactions: methods and protocols, 81–85. 10.1007/978-1-0716-3327-4_7 37450138

[B63] Gray W. M. Ostin A. Sandberg G. Romano C. P. Estelle M. (1998). High temperature promotes auxin-mediated hypocotyl elongation in arabidopsis. Proc. Natl. Acad. Sci. U. S. A. 95, 7197–7202. 10.1073/pnas.95.12.7197 9618562 PMC22781

[B19] Heinemann B. Künzler P. Eubel H. Braun H.-P. Hildebrandt T. M. (2021). Estimating the number of protein molecules in a plant cell: protein and amino acid homeostasis during drought. Plant Physiol. 185, 385–404. 10.1093/plphys/kiaa050 33721903 PMC8133651

[B20] Heusel M. Bludau I. Rosenberger G. Hafen R. Frank M. Banaei‐Esfahani A. (2019). Complex‐centric proteome profiling by SEC‐SWATH‐MS. Mol. Syst. Biol. 15, e8438. 10.15252/msb.20188438 30642884 PMC6346213

[B21] Jain R. Jenkins J. Shu S. Chern M. Martin J. A. Copetti D. (2019). Genome sequence of the model rice variety KitaakeX. BMC Genomics 20, 905. 10.1186/s12864-019-6262-4 31775618 PMC6882167

[B22] Jumper J. Evans R. Pritzel A. Green T. Figurnov M. Ronneberger O. (2021). Highly accurate protein structure prediction with AlphaFold. Nature 596, 583–589. 10.1038/s41586-021-03819-2 34265844 PMC8371605

[B23] Khan M. Youn J.-Y. Gingras A.-C. Subramaniam R. Desveaux D. (2018). In planta proximity dependent biotin identification (BioID). Sci. Rep. 8, 9212. 10.1038/s41598-018-27500-3 29907827 PMC6004002

[B24] Kim D. I. Jensen S. C. Noble K. A. Kc B. Roux K. H. Motamedchaboki K. (2016). An improved smaller biotin ligase for BioID proximity labeling. Mol. Biol. Cell 27, 1188–1196. 10.1091/mbc.E15-12-0844 26912792 PMC4831873

[B25] Larochelle M. Bergeron D. Arcand B. Bachand F. (2019). Proximity-dependent biotinylation mediated by TurboID to identify protein–protein interaction networks in yeast. J. Cell Science 132, 232249. 10.1242/jcs.232249 31064814

[B26] Lee M. (2023). Recent advances in deep learning for protein-protein interaction analysis: a comprehensive review. Molecules 28, 5169. 10.3390/molecules28135169 37446831 PMC10343845

[B64] Lei C. Zhou K. Zheng J. Zhao M. Huang Y. He H. (2023). AraPathogen2.0: an improved prediction of plant-pathogen protein-protein interactions empowered by the natural language processing technique. J. Proteome Res. 23, 494–499. 10.1021/acs.jproteome.3c00364 38069805

[B27] Liu Q. Zheng J. Sun W. Huo Y. Zhang L. Hao P. (2018). A proximity-tagging system to identify membrane protein–protein interactions. Nat. Methods 15, 715–722. 10.1038/s41592-018-0100-5 30104635

[B28] Maclean B. Tomazela D. M. Shulman N. Chambers M. Finney G. L. Frewen B. (2010). Skyline: an open source document editor for creating and analyzing targeted proteomics experiments. Bioinformatics 26, 966–968. 10.1093/bioinformatics/btq054 20147306 PMC2844992

[B29] Mair A. Xu S.-L. Branon T. C. Ting A. Y. Bergmann D. C. (2019). Proximity labeling of protein complexes and cell-type-specific organellar proteomes in Arabidopsis enabled by TurboID. Elife 8, e47864. 10.7554/eLife.47864 31535972 PMC6791687

[B30] Maruta N. Trusov Y. Botella J. R. (2016). “Yeast three-hybrid system for the detection of protein-protein interactions,” in Plant signal transduction: methods and protocols, 145–154. 10.1007/978-1-4939-3115-6_12 26577787

[B31] Matthes M. S. Yun N. Luichtl M. Büschges U. Fiesselmann B. S. Strickland B. (2025). Distinct domains of ENHANCER OF PINOID hold information for its polarization required for auxin-mediated cotyledon and flower development in Arabidopsis. PLoS Genetics 21, e1011217. 10.1371/journal.pgen.1011217 40549793 PMC12201645

[B32] Mcwhite C. D. Papoulas O. Drew K. Cox R. M. June V. Dong O. X. (2020). A pan-plant protein complex map reveals deep conservation and novel assemblies. Cell 181, 460–474.e414. 10.1016/j.cell.2020.02.049 32191846 PMC7297045

[B33] Miller J. P. Lo R. S. Ben-Hur A. Desmarais C. Stagljar I. Noble W. S. (2005). Large-scale identification of yeast integral membrane protein interactions. Proc. Natl. Acad. Sci. 102, 12123–12128. 10.1073/pnas.0505482102 16093310 PMC1189342

[B34] Montes C. Zhang J. Nolan T. M. Walley J. W. (2024). Single-cell proteomics differentiates Arabidopsis root cell types. New Phytol. 244, 1750–1759. 10.1111/nph.19923 38923440

[B35] Özmen B. Blaschek L. Ogden M. San Segundo M. Persson S. Zheng S. (2025). Proximity labeling techniques for protein–protein interaction mapping in plants. J. Biol. Chem. 301, 110501. 10.1016/j.jbc.2025.110501 40701246 PMC12359232

[B36] Pan J. You Z.-H. Li L.-P. Huang W.-Z. Guo J.-X. Yu C.-Q. (2022). Dwppi: a deep learning approach for predicting protein–protein interactions in plants based on multi-source information with a large-scale biological network. Front. Bioeng. Biotechnol. 10, 807522. 10.3389/fbioe.2022.807522 35387292 PMC8978800

[B37] Park T.-K. Kim T.-W. (2025). A brief guide to analyzing TurboID-Based proximity labeling-mass spectrometry in plants. Mol. Cells 48, 100236. 10.1016/j.mocell.2025.100236 40472971 PMC12264614

[B65] Petutschnig E. K. Pierdzig L. Mittendorf J. Niebisch J. M. Lipka V. (2024). A novel fluorescent protein pair facilitates FLIM-FRET analysis of plant immune receptor interaction under native conditions. J. Exp. Bot. 75, 746–759. 10.1093/jxb/erad418 37878766

[B66] Qiu Y. (2020). Regulation of PIF4-mediated thermosensory growth. Plant Sci. 297, 110541. 10.1016/j.plantsci.2020.110541 32563452

[B38] Rajczewski A. T. Blakeley‐Ruiz J. A. Meyer A. Vintila S. Mcilvin M. R. Van Den Bossche T. (2025). Data‐independent acquisition mass spectrometry as a tool for metaproteomics: interlaboratory comparison using a model microbiome. Proteomics 25, e202400187. 10.1002/pmic.202400187 40211604 PMC12696999

[B39] Ren H. Ou Q. Pu Q. Lou Y. Yang X. Han Y. (2024). Comprehensive review on bimolecular fluorescence complementation and its application in deciphering protein–protein interactions in cell signaling pathways. Biomolecules 14, 859. 10.3390/biom14070859 39062573 PMC11274695

[B40] Rosenthal S. M. Misra T. Abdouni H. Branon T. C. Ting A. Y. Scott I. C. (2021). A toolbox for efficient proximity-dependent biotinylation in zebrafish embryos. Mol. Cell. Proteomics 20, 100128. 10.1016/j.mcpro.2021.100128 34332124 PMC8383115

[B41] Roux K. J. Kim D. I. Raida M. Burke B. (2012). A promiscuous biotin ligase fusion protein identifies proximal and interacting proteins in mammalian cells. J. Cell Biol. 196, 801–810. 10.1083/jcb.201112098 22412018 PMC3308701

[B42] Sang T. Chen C.-W. Lin Z. Ma Y. Du Y. Lin P.-Y. (2024). DIA-based phosphoproteomics identifies early phosphorylation events in response to EGTA and mannitol in Arabidopsis. Mol. Cell. Proteomics 23, 100804. 10.1016/j.mcpro.2024.100804 38901673 PMC11325057

[B43] Sekar R. B. Periasamy A. (2003). Fluorescence resonance energy transfer (FRET) microscopy imaging of live cell protein localizations. J. Cell Biol. 160 (5), 629–633. 10.1083/jcb.200210140 12615908 PMC2173363

[B44] Senior A. W. Evans R. Jumper J. Kirkpatrick J. Sifre L. Green T. (2020). Improved protein structure prediction using potentials from deep learning. Nature 577, 706–710. 10.1038/s41586-019-1923-7 31942072

[B67] Sledzieski S. Singh R. Cowen L. Berger B. (2021). D-SCRIPT translates genome to phenome with sequence-based, structure-aware, genome-scale predictions of protein-protein interactions. Cell Syst. 12, 969–982.e6. 10.1016/j.cels.2021.08.010 34536380 PMC8586911

[B45] Struk S. Jacobs A. Sánchez Martín‐Fontecha E. Gevaert K. Cubas P. Goormachtig S. (2019). Exploring the protein–protein interaction landscape in plants. Plant, Cell Environ. 42, 387–409. 10.1111/pce.13433 30156707

[B46] Sun S. Yang X. Wang Y. Shen X. (2016). *In vivo* analysis of protein–protein interactions with bioluminescence resonance energy transfer (BRET): progress and prospects. Int. J. Mol. Sci. 17, 1704. 10.3390/ijms17101704 27727181 PMC5085736

[B47] Takano T. Wallace J. T. Baldwin K. T. Purkey A. M. Uezu A. Courtland J. L. (2020). Chemico-genetic discovery of astrocytic control of inhibition *in vivo* . Nature 588, 296–302. 10.1038/s41586-020-2926-0 33177716 PMC8011649

[B48] Tan B. Perron N. Guan Q. Zhu D. Singh Y. Dufresne C. (2025). Unraveling the molecular choreography of C3 to CAM transition in Mesembryanthemum crystallinum using phosphoproteomics. Plant J. 124, e70587. 10.1111/tpj.70587 41275434

[B49] Trinh C. S. Shrestha R. Conner W. C. Reyes A. V. Karunadasa S. S. Liu G. (2025). Mapping architecture of protein complexes in Arabidopsis using cross-linking mass spectrometry. BioRxiv. 10.1101/2025.04.28.651104 40777349 PMC12330729

[B50] Wanamaker S. A. Garza R. M. Macwilliams A. Nery J. R. Bartlett A. Castanon R. (2017). CrY2H-seq: a massively multiplexed assay for deep-coverage interactome mapping. Nat. Methods 14, 819–825. 10.1038/nmeth.4343 28650476 PMC5564216

[B51] Wee J. Wei G.-W. (2024). Evaluation of AlphaFold 3’s protein-protein complexes for predicting binding free energy changes upon mutation. J. Chem. Inf. Model. 64, 6676–6683. 10.1021/acs.jcim.4c00976 39116039 PMC11351016

[B52] Wen B. Hsu C. Shteynberg D. Zeng W. F. Riffle M. Chang A. (2025). Carafe enables high quality *in silico* spectral library generation for data-independent acquisition proteomics. Nat. Commun. 16 (1), 9815. 10.1038/s41467-025-64928-4 41198693 PMC12592563

[B53] Xing S. Wallmeroth N. Berendzen K. W. Grefen C. (2016). Techniques for the analysis of protein-protein interactions *in vivo* . Plant Physiol. 171, 727–758. 10.1104/pp.16.00470 27208310 PMC4902627

[B54] Ye R. Lin Z. Liu K.-H. Sheen J. Chen S. (2023). “Dynamic proximity tagging in living plant cells with pupylation-based interaction tagging,” in Protein-protein interactions: methods and protocols (Springer), 137–147. 10.1007/978-1-0716-3327-4_14 37450145

[B68] Yuan X. Deng H. Hu J. (2022). Constructing a PPI network based on deep transfer learning for protein complex detection. IEEJ Trans. Electr. Electron. Eng. 17, 436–444. 10.1002/tee.23524

[B55] Yue S. Xu P. Cao Z. Zhuang M. (2022). PUP-IT2 as an alternative strategy for PUP-IT proximity labeling. Front. Mol. Biosci. 9, 1007720. 10.3389/fmolb.2022.1007720 36250004 PMC9558124

[B56] Zhang Y. Gao P. Yuan J. S. (2010). Plant protein-protein interaction network and interactome. Curr. Genomics 11, 40–46. 10.2174/138920210790218016 20808522 PMC2851115

[B57] Zhang T. Schneider J. D. Lin C. Geng S. Ma T. Lawrence S. R. (2019). MPK4 phosphorylation dynamics and interacting proteins in plant immunity. J. Proteome Res. 18, 826–840. 10.1021/acs.jproteome.8b00345 30632760

[B58] Zhang B. Zhang Y. Liu J.-L. (2021). Highly effective proximate labeling in Drosophila. G3 11, jkab077. 10.1093/g3journal/jkab077 33724396 PMC8104946

[B59] Zhang K. Li Y. Huang T. Li Z. (2022). Potential application of TurboID-based proximity labeling in studying the protein interaction network in plant response to abiotic stress. Front. Plant Sci. 13, 974598. 10.3389/fpls.2022.974598 36051300 PMC9426856

[B60] Zhang Y. Ma X. Zhu M. Wang V.Y.-F. Guo J. (2025). Progress and prospects in FRET for the investigation of protein–protein interactions. Biosensors 15, 624. 10.3390/bios15090624 41002362 PMC12467375

[B61] Zheng S. Noack L. C. Khammy O. De Meyer A. Khan G. A. De Winne N. (2025). Pupylation-based proximity labeling reveals regulatory factors in cellulose biosynthesis in Arabidopsis. Nat. Commun. 16, 872. 10.1038/s41467-025-56192-3 39833163 PMC11747095

[B69] Zhou K. Lei C. Zheng J. Huang Y. Zhang Z. (2023). Pre-trained protein language model sheds new light on the prediction of arabidopsis protein-protein interactions. Plant Methods 19, 141. 10.1186/s13007-023-01119-6 38062445 PMC10704805

